# ’Twixt Tweedle-dum and Tweedle-dee

**Published:** 1890-07

**Authors:** 


'Twixt Tweedle-dum and Tweedle-dee.—The *Nashville Journal of Medicine and Surgery*, the organ of the president-elect of the American Medical Association, says that at the last meeting of the Tennessee Medical Society a resolution was introduced by Dr. J. D. Cole, of Newbern, in reference to the construction of that part of the code looking to consultation with homeopathic or other irregular practitioners. Commenting thereon it says: "There can be no doubt but that certain circumstances might be looked upon as mitigating the stringency of this section of the code, as for example the transference of a surgical case by an irregular to a surgeon, and it would seem that at times such a combination of circumstances have been taken advantage of, surgeons meeting and continuing in consultation with irregulars in cases entrusted to the former by the latter." Wherein does this position of the *Journal* differ from the much opposed "emergency" clause of the New York code, which led to the exclusion of the Medical Society of that State from the American Medical Association?—*Medical Standard, Chicago.* Echo answers "Wherein."

